# 

**Published:** 1892-08

**Authors:** 


					﻿Vol XII.
AUGUST, 1892.
NO. 8.
THE
HOMEOPATHIC PHYSICIAN,
A Monthly Journal of Homoeopathic Materia Medica
and Clinical Medicine.
“ He is a freeman whom truth makes free, and all are slaves beside."
“Seek the Truth: come whence it may, cost what it will."
EDITED AND PUBLISHED BY
WALTER M. JAMES, M. LX
PHILADELPHIA :
No. 1125 SPRUCE STREET.
LONDON:
ALFRED HEATH A CO., Bole Agents bob Great Britain,
114 Ebury Street, Eaton Square.
Yearly Subscription, $2.50. invariably in advance. Single numbers, 25 ets.
Entered at the Philadelphia Peet-Offioe an Seoond-Claae Mail Matter.
				

